# Efficacy of PI3K/AKT/mTOR pathway inhibitors for the treatment of advanced solid cancers: A literature-based meta-analysis of 46 randomised control trials

**DOI:** 10.1371/journal.pone.0192464

**Published:** 2018-02-06

**Authors:** Xuan Li, Danian Dai, Bo Chen, Hailin Tang, Xiaoming Xie, Weidong Wei

**Affiliations:** Department of Breast Oncology, Sun Yat-Sen University Cancer Center, State Key Laboratory of Oncology in South China, Collaborative Innovation Center for Cancer Medicine, Guangzhou, China; Queen Mary University of London, UNITED KINGDOM

## Abstract

**Background:**

The phosphatidylinositol-3- kinase (PI3K)/AKT/mammalian target of rapamycin (mTOR) pathway (PI3K/AKT/mTOR pathway) plays a key role in cancer. We performed this meta-analysis to assess the clinical effect of using PI3K/AKT/mTOR pathway inhibitors on advanced solid tumours.

**Methods:**

All the randomised controlled trials (RCT) that compared the therapy with PI3K/AKT/mTOR pathway inhibitors with other therapies were included. The main end-point was progression-free survival (PFS); other end-points included overall survival (OS) and objective response rate (ORR). A subgroup analysis was performed mainly for PFS.

**Results:**

In total, 46 eligible RCT were included. The pooled results showed that PI3K/AKT/mTOR pathway inhibitor-based regimens significantly improved the PFS of patients with advanced solid tumours (hazard ratios (HR) = 0.79; 95% confidence intervals (CI): 0.71–0.88) and PI3K pathway mutations (HR = 0.69; 95% CI: 0.56–0.85). All single PI3K/AKT/mTOR pathway inhibitor therapies were compared with other targeted therapies (HR = 0.99; 95% CI: 0.93–1.06) and dual targeted therapies, including PI3K/AKT/mTOR pathway inhibitors and other targeted therapies (HR = 1.04; 95% CI: 0.62–1.74), which showed no significant differences in the PFS. Additional PI3K/AKT/mTOR pathway inhibitors showed no advantage with respect to the OS (HR = 0.98; 95% CI: 0.90–1.07) or ORR (risk ratio (RR) = 1.02; 95% CI: 0.87–1.20).

**Conclusion:**

Our meta-analysis results suggest that the addition of the PI3K pathway inhibitors to the therapy regiment for advanced solid tumours significantly improves PFS. The way that patients are selected to receive the PI3K pathway inhibitors might be more meaningful in the future.

## Introduction

The PI3K/AKT/mTOR pathway plays a key role in the promotion of cell survival and proliferation in cancers[1, 2], and elevated PI3K pathway signalling seems to be a hallmark of cancer. Three classes of PI3K enzymes (Class I, II, III PI3K) are expressed in human cells, and the lipid product of class I PI3Ks activates the downstream kinase AKT (AKT1, AKT2, AKT3). The mTOR protein has two cellular complexes (mTORC1 and mTORC2), one of which (mTORC1) is a key node in cell growth that can be activated by PI3K/AKT signals or signals from other pathways[3, 4]. Activating mutations in the PI3K pathway are commonly found in solid cancers; in advanced cancers, this mutation rate can increase by 30% -60% in different tumour types, such as breast cancer, gastric cancer and colorectal cancer[5–8].

In solid cancers, preclinical tests have shown that a hyperactive PI3K pathway treated by PI3K or mTOR inhibitors results in the restoration of sensitivity of cancer cell lines to restore sensitivity to hormone therapy, chemotherapy or other targeted therapies[9–12]. With the discovery of the tumourigenesis function of the PI3K pathway, many PI3K pathway inhibitors have been generated and tested in clinical trials. Many phase I trials of PI3K pathway inhibitors have assessed their anti-tumour activity alone or combined with other therapies, but the dose-limited toxicities have still halted some trials early and have prevented further testing[13–15]. Those phase II and III trials that have tested the anti-tumour effects of PI3K pathway inhibitors are disputed, and some actual clinical results are apparently lower than expected. Multiple pathways activated together with the PI3K pathway, mutations in specific genes and dose-limited toxicities prevent drugs from achieving the best inhibitory effects and are the major factors that may weaken the effects of PI3K inhibitors effects. The results from some well-designed clinical trials that have attempted to solve the aforementioned problems must be summarized.

In this study, we have analyzed the RCTs of PI3K/AKT/mTOR pathway inhibitors to assess their efficacy in all advanced solid cancers and whether they exhibit more efficient anti-tumour properties when combined with other targeted regimens or in cancers with PI3K mutations.

## Materials and methods

### Data retrieval strategies

We have conducted this meta-analysis in accordance with the PRISMA statement (S1 Table). Relevant publications from PubMed, Web of Science and Embase were identified. The following medical subject heading terms were searched for: ‘Tumours OR Neoplasm OR Cancer OR Solid tumour’ AND ‘PI3K inhibitor OR AKT inhibitor OR mTOR inhibitor OR PI3K/AKT/mTOR inhibitor OR PI3K/mTOR inhibitor’ AND ‘random OR clinical OR control OR randomised control trial OR RCT’. We have also manually searched for the drug names of PI3K pathway inhibitors provided by Fruman[2]and crosschecked the references to complete the results from the searches of the databases for publications up to September 01, 2017. Only those studies that definitively indicated that their results were from a phase II or III randomized controlled trial (RCT) and those that enrolled more than 10 patients in each arm were used. When utilizing results from the same trial were considered, we screened for the most complete and recent data.

### Inclusion criteria

The following study inclusion criteria were used: (1) participants with advanced or metastatic solid tumours; (2) a clearly defined therapy with PI3K/AKT/mTOR pathway inhibitors in the experimental arm; (3) inclusion of placebo or other anti-tumour agents but not PI3K/AKT/mTOR pathway inhibitors in the control arm; and (4) the outcomes of progression-free survival (PFS), time to progression (TTP) and overall survival (OS) expressed as hazard ratios (HRs) or objective response rates (ORRs) could be extracted. The exclusion criteria were as follows: (1) studies including non-solid tumours; (2) insufficient data; (2) the number of patients in an arm was < 10; and (3) non-randomised studies.

### Data extraction

Two authors (XL and BC) independently screened and selected the data independently. Any disputed results were reviewed by a third author (DD). Relevant data included the name of the first author, publication year, trial name (if available), tumour types, the trial phase, the chemical properties of the experimental and control arms, the number of subjects in each arm, specific protocols, survival outcomes of PFS (as HRs), TTP and OS and the number of patients who experienced a complete or partial response in each arm. Considering the definition of TTP, we included the TTP results as part of in the PFS. For each trial, an arm was considered the experimental arm if it included a treatment with PI3K/AKT/mTOR pathway inhibitors, while the arm with placebo or other anti-tumour agents was considered the control. A PI3K mutational analysis was performed by PCR or gene sequencing. The five-item Jadad scale, which accounts for randomisation, blinding and withdrawals or dropouts, was used to assess the quality of each study[16]; scores ranged from 0 to 5.

### Statistical analysis

The Q-test and the I^2^ statistic were used to assess statistical heterogeneity. I^2^ values lower than 25% and P > 0.1 were considered to indicate low heterogeneity according to a fixed-effects model (Mantel-Haenszel method). I^2^ values higher than 50% or I^2^ < 50% but P < 0.1 were considered to indicate moderate or high heterogeneity, according to a random-effects model. Survival outcomes, including OS and PFS, were expressed as HRs with 95% confidence intervals (CIs) for each study. The RRs with 95% CIs were calculated as the result of the dichotomous variable of the objective response rate for each study. Subgroup analyses were performed for the different tumour types, treatment protocols and gene statuses. Egger’s test was used to assess the publication bias by Stata and P>|t| > 0.05 indicates no significant publication bias. All statistical tests were two-sided, and the value of P < 0.05 was considered significant. The statistical tests were mostly performed primarily in Revman 5.3.

## Results

**Fig 1:**
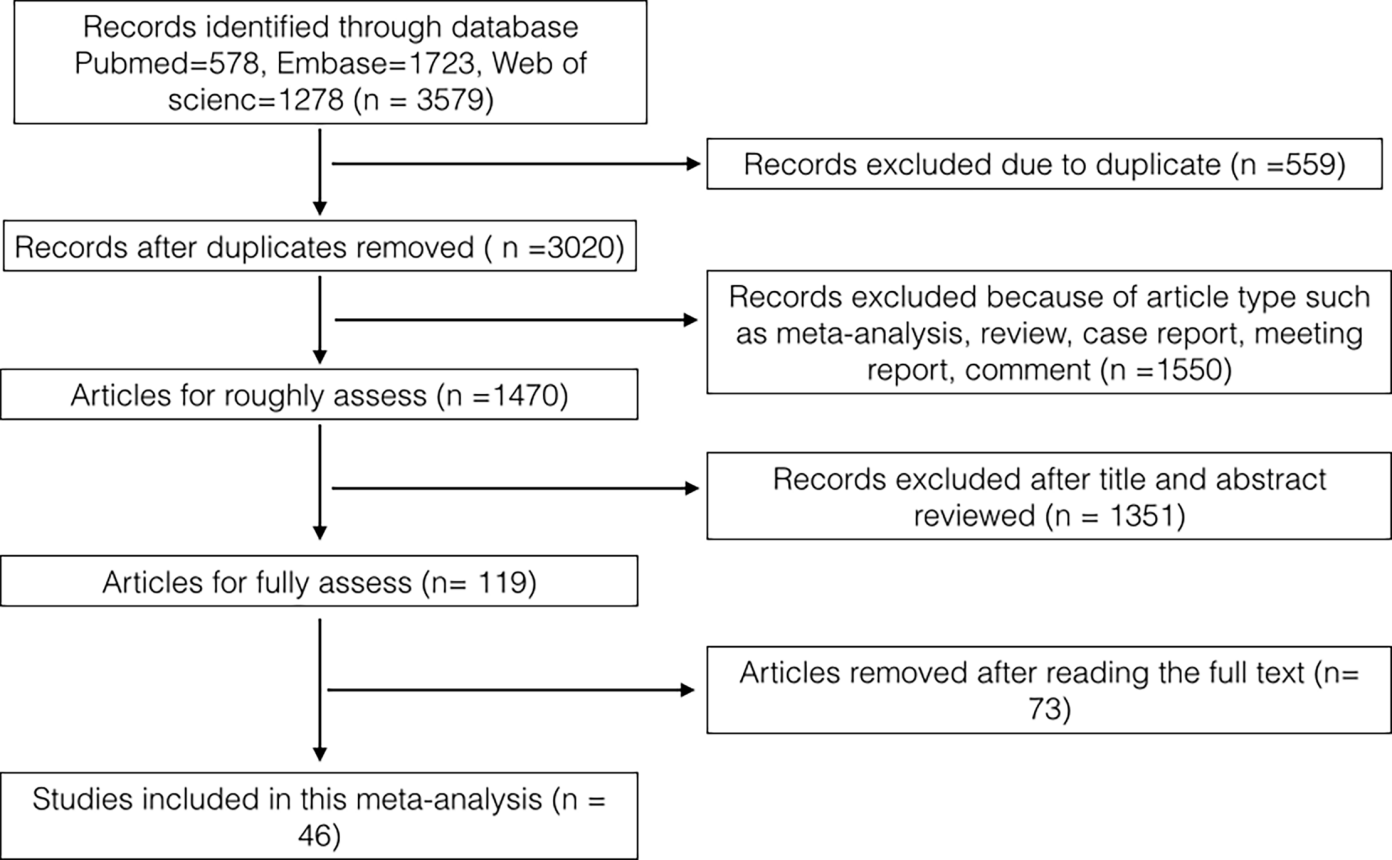
The flowchart of selection process.

This study found 3579 potentially relevant articles, but 559 studies were excluded because they were duplicate reports. After a carefully review of the remaining studies, the full texts of 46 RCT studies were included in the final analysis (Fig 1). All included studies focused on advanced or metastatic solid tumours. Twelve studies focused on breast cancer[17–28], 13 on renal cancer[29–41], 4 on lung cancer[42–45], 4 on neuroendocrine tumors[46–49], 3 on gastrointestinal cancer[50–52], 3 on head and neck squamous cell cancer[53–55], 2 on sarcomas[56, 57], 1 on liver cancer[58], 1 on pancreatic cancer[59], 1 on endometrial cancer[60], 1 on glioblastoma[61] and 1 on melanoma[62].The basic characteristics of the studies are outlined in Table 1. A total of 15511 cases were included in the meta-analysis, namely, 8478 cases in the experimental groups and 7033 cases in the control groups. Nineteen phase III RCT studies and 27 phase II RCT studies were analysed. A total of 32 studies reported mTOR inhibitors, 9 reported PI3K inhibitors, 4 reported AKT inhibitors and 1 reported PI3K/AKT/mTOR pathway inhibitors. The Egger’ s test results were P > |t| = 0.230 for PFS and P > |t| = 0.957 for OS showing no significant publication bias in this analysis. The Jadad score of the studies included in the meta-analysis ranged from 4 to 5. Thus, all studies were of good quality (Table 1 and S2 Table).

**Table 1:** Characteristics of the included studies.

Study	Publish year	Tumor type	Trial phase	Experiment arm targeted reagents type	Control arm or experiment arm combined targeted reagents type	General protocol	Patients numbers in experimental arm	Patients numbers in control arm	Primary end-point	Other end-point	Reported the PI3K mutant data (yes/no)	Jaded Score
Andre (BOLERO-3)	2014	Breast cancer	III	mTORC1	HER2	Everolimus+ Vinorelbine+ trastuzumab vs placebo+Vinorelbine+ trastuzumab	284	285	PFS	NA	yes	5
Bachelot (GINECO)	2012	Breast cancer	II	mTORC1	NA	Everolimus+ tamoxifen vs tamoxifen	54	57	CBR	TTP, OS	no	4
Baselga (BOLERO-2)	2012	Breast cancer	III	mTORC1	NA	Everolimus + exemestane vs placebo+ exemestane	485	239	PFS	OS	no	5
Baselga (BELLE-2)	2017	Breast cancer	III	Pan-PI3K	NA	Buparlisib+ fulvestrant vs placebo+ fulvestrant	576	571	PFS	NA	yes	5
Baselga*	2017	Breast cancer	II	mTORC1	NA	Ridaforolimus+ dalotuzumab+ exemestane vs exemestane	29	33	PFS	OS	no	4
Hurvitz (BOLERO-1)	2015	Breast cancer	III	mTORC1	HER2	Everolimus+ Trastuzumab+ Paclitaxel vs placebo+ Trastuzumab+ Paclitaxel	480	239	PFS	NA	no	5
Kim (LOTUS)	2017	Breast cancer	II	AKT	NA	Ipatasertib+ paclitaxel vs placebo+ paclitaxel	62	62	PFS	NA	yes	5
Krop (FERGI)	2016	Breast cancer	II	Pan-PI3K	NA	Pictilisib+ fulvestrant vs placebo+ fulvestrant	89	79	PFS	NA	yes	5
Martin (BELLE-4)	2016	Breast cancer	III	Pan-PI3K	NA	Buparlisib+ paclitaxel vs placebo+ paclitaxel	207	209	PFS	NA	yes	5
Vuylsteke (PEGGY)	2016	Breast cancer	II	Pan-PI3K	NA	Pictilisib+ paclitaxel vs placebo+ paclitaxel	91	92	PFS	NA	yes	4
Wolff (HORIZON)	2013	Breast cancer	III	mTORC1	NA	Temsirolimus+letrozole vs placebo+letrozole	555	555	PFS	OS	no	5
Yardley	2015	Breast cancer	II	mTORC1	VEGF inhibitor	Everolimus +Paclitaxel+ Bevacizumab vs placebo+ Paclitaxel+ Bevacizumab	56	57	PFS	OS	no	4
Armstrong (ASPEN)	2016	Renal cell cancer	II	mTORC1	VEGFR inhibitor	everolimus vs sunitinib	57	51	PFS	OS	no	4
Choueiri (METEOR)	2016	Renal cell cancer	III	mTORC1	VEGFR inhibitor	Everolimus vs cabozantinib	328	330	PFS	OS	no	4
Cirkel (ROPETAR)	2016	Renal cell cancer	II	mTORC1	VEGFR inhibitor	Everolimus+ pazopanib vs pazopanib	52	49	PFS	NA	no	4
Dutcher#a; b	2009	Renal cell cancer(a: clear cell cancer; b: no clear cell cancer)	III	mTORC1	NA	Temsirolimus vs interferon	a: 169; b: 37	a: 170; b: 18	OS	PFS	no	4
Flaherty#a; b; c (ECOG2804)	2015	Renal cell cancer	II	mTORC1	VEGF inhibitors	(a) Bevacizumab plus temsirolimus vs bevacizumab alone (b) Bevacizumab plus temsirolimus vs bevacizumab plus sorafenib (c) Sorafenib plus temsirolimus vs bevacizumab plus sorafenib	a: 80; b: 80; c: 84	a: 84; b: 83; c: 83	PFS	NA	no	4
Hudes#a; b	2007	Renal cell cancer	III	mTORC1	NA	(a) Temsirolimus vs interferon (b) Temsirolimus+ interferon vs interferon	a: 210; b: 209	a&b: 207	OS	PFS	no	4
Hutson	2013	Renal cell cancer	III	mTORC1	VEGF inhibitor	Temsirolimus vs sorafenib	259	253	PFS	OS	no	4
Motzer (RECORD-1)	2010	Renal cell cancer	III	mTORC1	NA	Everolimus vs placebo	277	139	PFS	OS	no	5
Motzer (RECORD-3)	2014	Renal cell cancer	II	mTORC1	VEGF inhibitor	Everolimus vs sunitinib	238	233	PFS	NA	no	4
Motzer	2015	Renal cell cancer	III	mTORC1	PD-1 inhibitor	Everolimus vs Nivolumab	410	411	OS	PFS	no	4
Negrier (TORAVA)	2011	Renal cell cancer	II	mTORC1	VEGF inhibitor	Temsirolimus+ bevacizumab vs interferon alfa + bevacizumab	88	40	PFS	NA	no	4
Rini (INTORACT)	2013	Renal cell cancer	III	mTORC1	VEGF inhibitor	Temsirolimus+ bevacizumab vs IFN+ bevacizumab	400	391	PFS	OS	no	4
Tannir	2015	Renal cell cancer	II	mTORC1	VEGFR inhibitor	Temsirolimus vs sunitinib	35	33	PFS	NA	no	4
Besse	2014	Lung cancer	II	mTORC1	EGFR inhibitor	Everolimus+ erlotinib vs erlotinib	66	67	DCR	PFS; OS	no	4
Levy	2014	Lung cancer	II	Pan-PI3K	NA	PX-866+ docetaxel vs docetaxel	48	47	PFS	OS	no	4
Papadimitrakopoulou (BATTLE-2)	2016	Lung cancer	II	AKT	EGFR inhibitor	MK-2206+erlotinib vs erlotinib	42	22	DCR	PFS; OS	no	4
Socinski (TAX 326)	2010	Lung cancer	II	AKT	NA	Enzastaurin+ carboplatin vs carboplatin	72	74	TTP	OS	no	4
Zhu (EVOLVE-1)	2014	Liver cancer	III	mTORC1	NA	Everolimus vs placebo	362	184	OS	TTP	no	5
Bendell	2011	Colorectal Cancer	II	PI3K/ Akt/mTOR signaling inhibitor	NA	Perifosine+ capecitabine vs placebo + capecitabine	20	18	TTP	OS	no	5
Bowles	2016	Colorectal Cancer	II	Pan-PI3K	EGFR inhibitor	PX-866 + cetuximab vs placebo+ cetuximab	42	38	PFS	OS	no	4
Ohtsu (GRANITE-1)	2013	Gastric cancer	III	mTORC1	NA	Everolimus vs placebo	439	217	OS	PFS	no	5
Jimeno	2015	Head and neck squamous cell cancer	II	Pan-PI3K	EGFR inhibitor	PX-866+cetuximab vs cetuximab	42	41	PFS	OS	no	4
Jimeno	2016	Head and neck squamous cell cancer	II	Pan-PI3K	NA	PX-866 + docetaxel vs docetaxel	42	43	PFS	OS	no	4
Soulieres (BERIL-1)	2017	Head and neck squamous cell cancer	II	Pan-PI3K	NA	Buparlisib + paclitaxel vs placebo + paclitaxel	79	79	PFS	OS	no	5
Rachards	2011	Pancreatic cancer	II	AKT	NA	Enzastaurin+ gemcitabine vs gemcitabine	86	44	OS	PFS	no	4
Pavel (RADIANT-2)	2011	Neuroendocrine tumours	III	mTORC1	NA	Everolimus + octreotide LAR vs placebo+ octreotide LAR	216	213	PFS	OS	no	5
Yao (RADIANT-3)	2011	Neuroendocrine tumours	III	mTORC1	NA	Everolimus vs placebo	207	203	PFS	OS	no	5
Yao (RADIANT-3)	2014	Neuroendocrine tumours	II	mTORC1	NA	Everolimus vs placebo	44	35	PFS	OS	no	5
Yao (RADIANT-4)	2016	Neuroendocrine tumours	III	mTORC1	NA	Everolimus vs placebo	205	97	PFS	OS	no	5
Eroglu	2015	Sarcoma	II	mTORC1	RAF/MEK/ERK (MEK1) inhibitor	Temsirolimus + selumetinib vs selumetinib	35	34	PFS	NA	no	4
Demetri	2013	Sarcoma	III	mTORC1	NA	Redaforolimus vs placebo	347	364	PFS	OS	no	5
Oza	2015	Endometrial cancer	II	mTORC1	NA	Ridaforolimus vs progestin or chemotherapy	64	66	PFS	OS	no	4
Wick (EORTC 26082)	2016	Glioblastoma	II	mTORC1	NA	Temsirolimus vs temozolomide	56	55	OS	PFS	no	4
Margolin (S0438)	2012	Melanoma	II	mTORC1	VEGFR inhibitor and/or RAF/MEK/ERK inhibitor	Temsirolimus+ sorafenib vs tipifarnib+ sorafenib	63	39	PFS	OS	no	4

Abbreviation: NR: not reported; NA: not available; PFS: progression-free survival; OS: overall survival; IFN: interferon; ORR: objective response rate; TTP: time to progression; CBR: clinical benefit rate; DCR: Disease control rate; EGFR: epidermal growth factor receptor; VEGFR: vascular endothelial growth factor receptor; mTORC1: mammalian target of rapamycin complex 1.

* Reported a different trial by the previous author in the same year.

# Reported more than one comparation in a trial. Lowercase letter a, b,c means different trial arm in the same study.

### Progression-free survival

**Fig 2:**
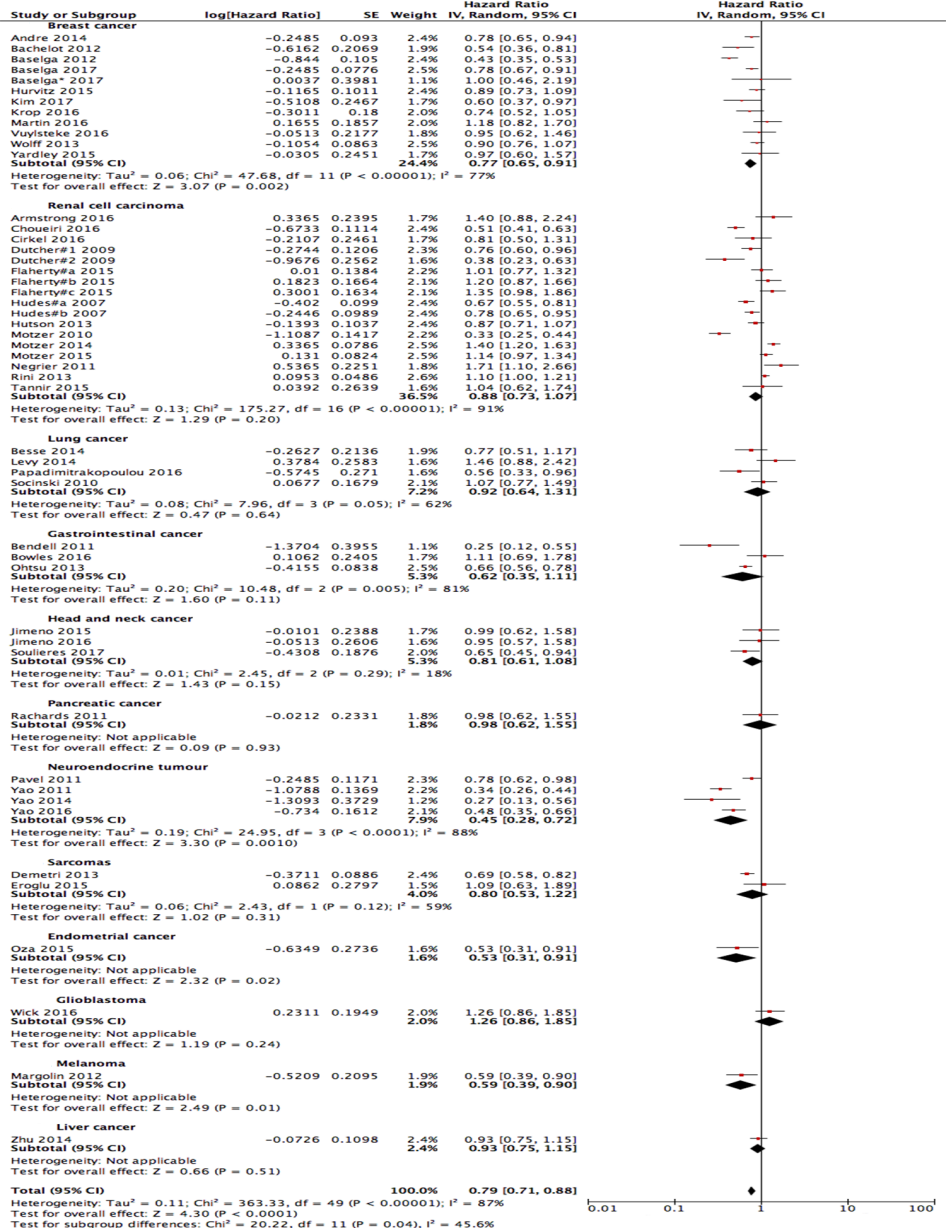
Forest plots of hazard ratios (HRs). Progression-free survival (PFS) comparing PI3K/AKT/mTOR inhibitors with the control arm. A random-effects model was used.

**Fig 3:**
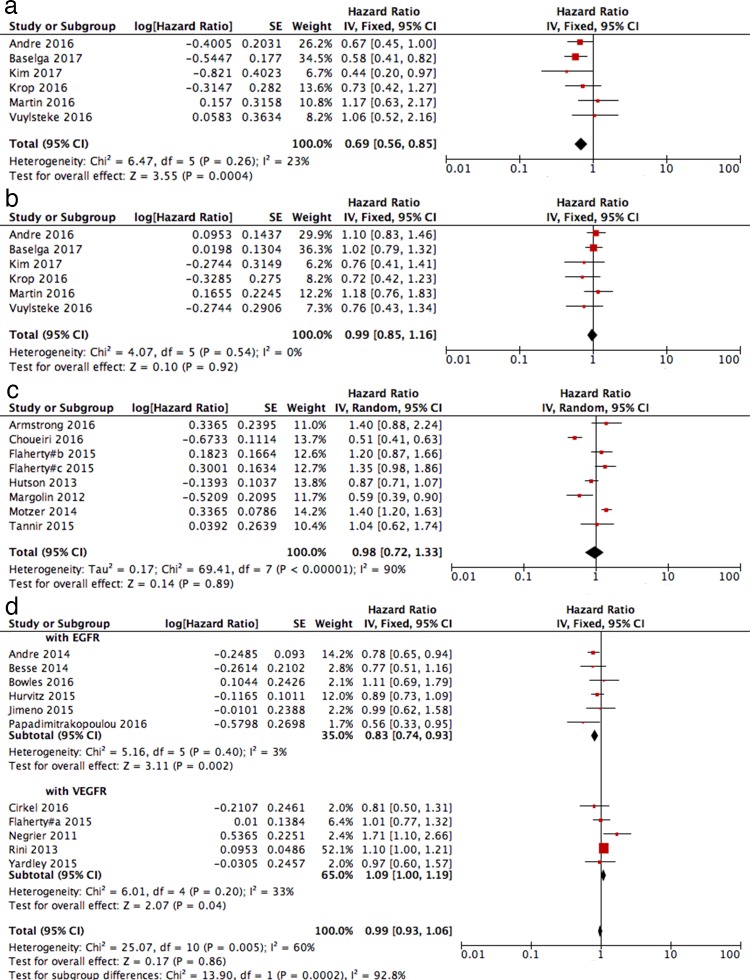
Subgroup analyses for PFS. Forest plots of hazard ratios (HRs) for PFS when PI3K/AKT/mTOR inhibitors were compared with the control arm. (a) PI3K mutant cancer; (b) PI3K non-mutant cancer; (c) single PI3K/AKT/mTOR inhibitor compared with other target therapy (VEGF/VEGF receptor inhibitors); (d) PI3K/AKT/mTOR inhibitors combined with another targeted reagent therapy compared with single targeted therapy without PI3K/AKT/mTOR inhibitors.

All 46 studies reported PFS data, and 4 of these reported TTP results. Three studies reported more than 1 comparison. Thus, 50 pairs of control arms were included in this analysis. The pooled analysis showed an improvement in the PFS when using the PI3K/AKT/mTOR pathway inhibitor-based therapies were used, but with high heterogeneity (HR = 0.79; 95% CI: 0.71–0.88; I^2^ = 87%, random-effects model; Fig 2). A subgroup analysis showed that PI3K/AKT/mTOR pathway inhibitor-based therapy significantly improved the PFS in all solid tumour types except glioblastoma. Significant differences in the PFS between the experimental and control arms were found in breast cancer, neuroendocrine tumours, endometrial cancer and melanoma. An analysis of the results according to the type of PI3K/AKT/mTOR pathway inhibitors showed that mTOR inhibitors, pan-PI3K inhibitors and AKT inhibitors all improved the PFS (data not shown). Six studies reported PFS data on patients with or without PI3K pathway mutations, and 1 of them included the pooled results of 2 RCT studies. The use of PI3K/AKT/mTOR pathway inhibitor-based therapies improved the PFS of patients with PI3K pathway mutations, as shown by the significant differences in PFS (HR = 0.69; 95% CI: 0.56–0.85; I^2^ = 23%, fixed-effects model; Fig 3 (A)). The PFS of patients without PI3K pathway mutations improved slightly, albeit with no significant differences (HR = 0.99; 95% CI: 0.85–1.16; I^2^ = 0%, fixed-effects model; Fig 3 (B)). Eight studies compared PI3K/AKT/mTOR pathway inhibitors with other targeted therapies, all of which were VEGF/VEGF receptor inhibitors. A subgroup analysis revealed no significant differences in the PFS of these patients (HR = 0.98; 95% CI: 0.72–1.33; I^2^ = 90%, random-effects model; Fig 3 (C)). Six studies compared dual-targeted therapies including PI3K/AKT/mTOR pathway inhibitors and EGFR inhibitors with EGFR inhibitors alone. The pooled results showed significant improvement as a result of dual-targeted therapies with an HR = 0.83 (95% CI: 0.74–0.93; I^2^ = 3%, fixed-effects model Fig 3 (D)). However, the comparison of dual-targeted therapies including PI3K/AKT/mTOR pathway inhibitors and VEGF/VEGF receptor inhibitors with VEGF/VEGF receptor inhibitors alone showed a poorer PFS for patients treated with dual-targeted therapies (HR = 1.09; 95% CI: 1.00–1.19; I^2^ = 33%, fixed-effects model; Fig 3 (D)). The pooled results of dual-targeted therapies including PI3K/AKT/mTOR pathway inhibitors compared with single-targeted therapies showed no significant differences and high heterogeneity, which may be partly due to the drugs used together with the PI3K/AKT/mTOR pathway inhibitors (HR = 0.99; 95% CI: 0.93–1.06; I^2^ = 60%; Fig 3 (D)).

### Overall survival

**Fig 4:**
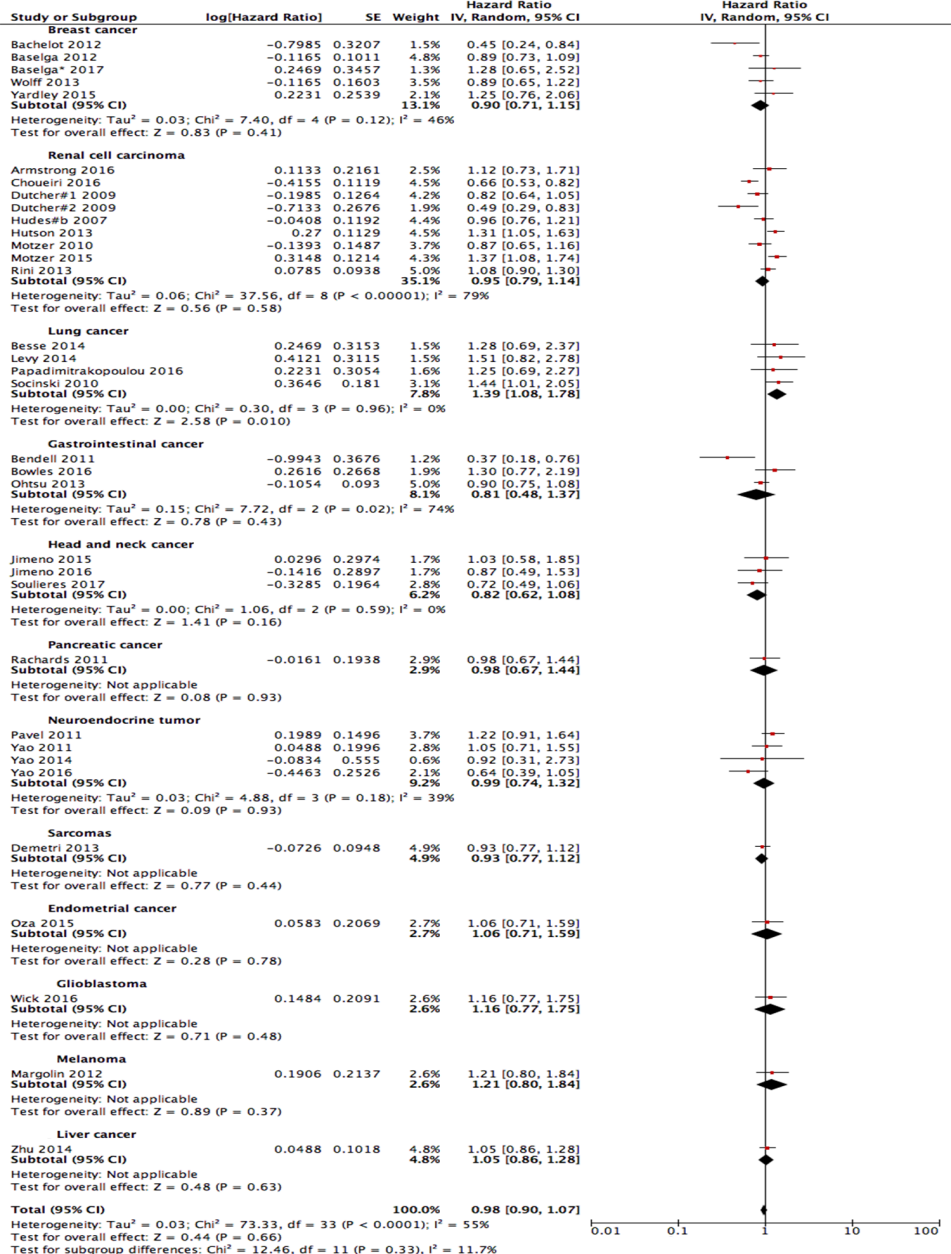
Forest plots of hazard ratios (HRs). Overall survival (OS) when PI3K/AKT/mTOR inhibitors were compared with the control arm. The random-effects model was used.

Data were obtained on the OS of 34 compared arms. The pooled analysis of these studies showed that PI3K/AKT/mTOR pathway inhibitor-based therapies slightly improved the OS of patients with solid tumours compared with that of the control arms, but differences were not significant (HR = 0.98; 95% CI: 0.90–1.07; I^2^ = 55%, random-effects model; Fig 4). A subgroup analysis showed that PI3K/AKT/mTOR pathway inhibitor-based therapies improved the OS of the patients with breast cancer, renal cancer, gastrointestinal cancer, head and neck squamous cell cancer, pancreatic cancer, neuroendocrine tumour and sarcomas but the differences were not statistically. In other types of cancer, the PI3K/AKT/mTOR pathway inhibitor-based therapies apparently failed to improve the OS.

### Objective response rate

**Fig 5:**
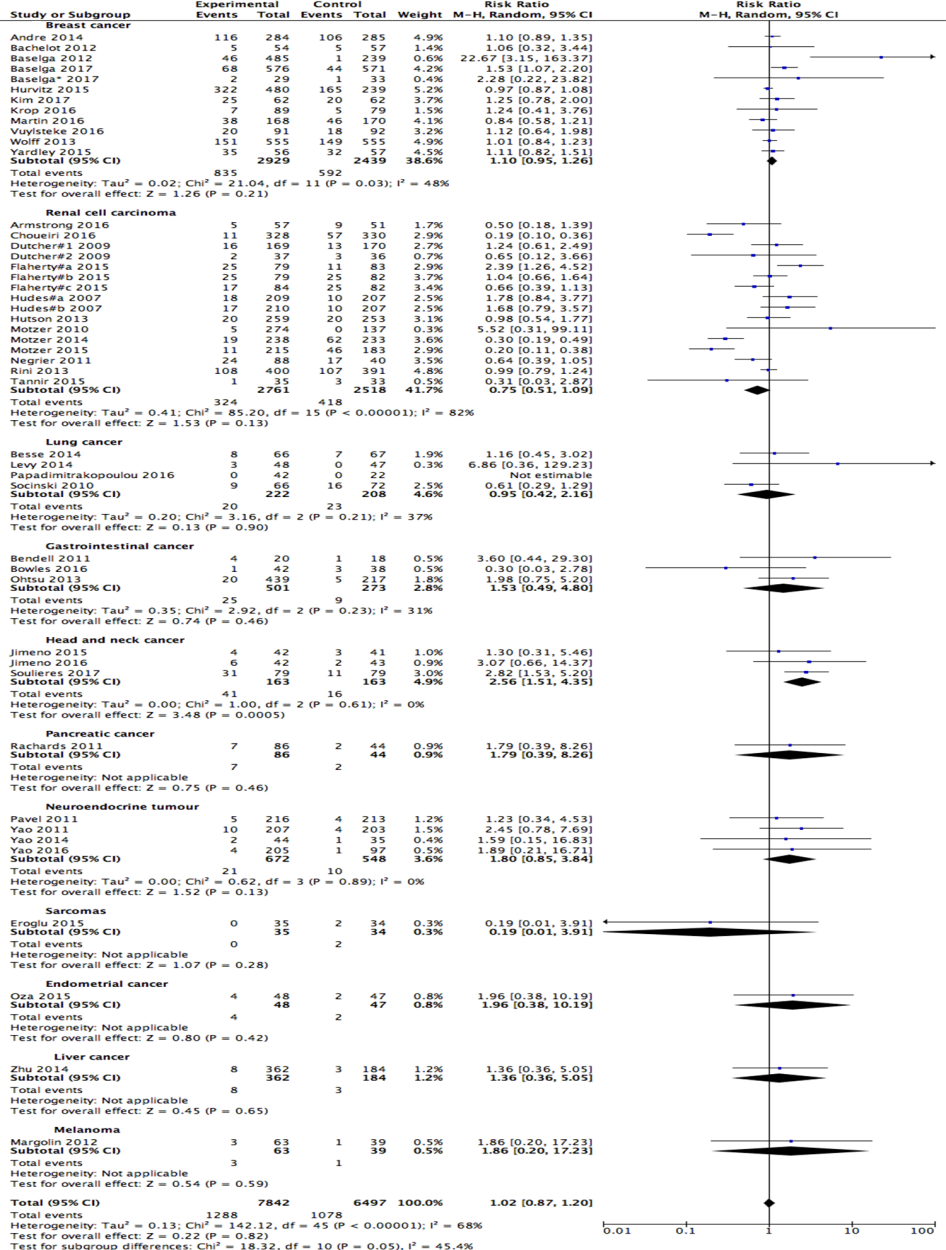
Forest plots of the risk ratio (RR) for the objective response rate (ORR) comparing PI3K/AKT/mTOR inhibitors with the control arm. A random-effects model was used.

An objective response rate was found in 1288/7842 (16.4%) and 1078/6497 (16.6%) patients from the experimental and control arms, respectively. The risk ratio (RR) pooled from combined trials using the Mantel-Haenszel method was 1.02 (95% CI: 0.87–1.20; I^2^ = 68%, random-effects model; Fig 5), which thus favours the therapeutic regimen without PI3K/AKT/mTOR pathway inhibitors. The ORR of renal cancer, lung cancer and sarcomas favoured the experimental arm, although they did not all reach statistical significance.

### Discontinued rate

The use of PI3K/AKT/mTOR inhibitors was associated with a higher rate of discontinuation because of toxic and adverse effects (OR = 2.16; 95% CI: 1.59–2.95; I^2^ = 72%, random-effects model; S1 Fig). A subgroup analysis according to PI3K/AKT/mTOR inhibitors showed that the patients who received a therapy regimen consisting of mTOR inhibitors (OR = 2.35; 95% CI: 1.66–3.31; I^2^ = 76%, random-effects model; S1 Fig) or AKT inhibitors (OR = 2.61; 95% CI: 1.06–6.45; I^2^ = 0%, random-effects model; S1 Fig) showed more than a 2-fold ratio of study discontinuation because of adverse events; these differences were statistically significant. The use of pan-PI3K inhibitors also resulted in a higher ratio of adverse events, which led to study discontinuation, but the differences were not statistically significant (OR = 1.47; 95% CI: 0.53–4.13; I^2^ = 73%, random-effects model; S1 Fig).

## Discussion

This systematic review and meta-analysis, which included 46 randomized controlled trials with a total of 15511 patients and more than 100 arms, was conducted to fully assess the effect of PI3K pathway inhibitors on solid tumours. Our analysis showed that the addition of PI3K pathway inhibitors significantly improves the PFS of subjects with in advanced solid cancers, although their efficacy differed among tumour types. We found that most trials focused on breast cancer, renal cancer, lung cancer, gastrointestinal cancer, head and neck squamous cell cancer and neuroendocrine tumours. Our analysis results suggest that the PI3K/AKT/mTOR inhibitors added to the therapy regimen significantly improved the PFS especially among patients with breast cancer and neuroendocrine tumours. Patients with mutations in the PI3K pathway may benefit more from treatment with PI3K pathway inhibitors than patients without mutations based on the PFS. The pooled results showed no improvement in OS inhibitors or in ORR as a result of the treatment of advanced solid tumours with PI3K pathway inhibitors.

In this study, we focused on PI3K pathway inhibitors, particularly mTORC1 inhibitors, Pan-PI3K inhibitors and a few AKT and multiple-target inhibitors (Table 1). The mTOR pathway functions primarily through the PI3K/AKT pathway to activate the tumour cells; members of the PI3K pathway family are frequently altered in human cancers, which leads to cell survival and proliferation, metastasis and activation of some secretion functions[2, 63]. The inhibition of one or more markers in this pathway can induce anti-tumour effects in preclinical studies[64]. Some meta-analyses studies have reported the treatment of some tumours with everolimus (a mTOR inhibitor) and found it to be associated with a lower risk of poor PFS, but no significant differences were observed in any of the tests[65, 66]. In this study, the PFS-related benefit was highest when mTOR inhibitors (HR 0.78; 95% CI: 0.68–0.89) were used, followed by AKT inhibitors (HR 0.81; 95% CI: 0.59–1.11) and pan-PI3K inhibitors (HR 0.91; 95% CI: 0.77–1.06). The direct comparison between AKT inhibitors and mTORC dual inhibitors or pan-PI3K and mTOR dual inhibitors with isolated mTORC1 inhibitors in some phase II trials showed no improvement in PFS[67–69], most likely because mTORCI is located at the centre of the PI3K/AKT/mTOR pathway, and parallel, but not linear, pathway inhibition may increase the clinical efficacy.

The comparison between PI3K pathway inhibitors and other targeted inhibitors, such as PD-1 inhibitors, MAPK pathway inhibitors and VEGFR inhibitors, showed no benefits in PFS. In our subgroup analysis, single mTOR inhibitors compared with VEGFR inhibitors resulted in a similar risk in terms of PFS. Many axes or molecular targets were activated along with the PI3K pathway, such as the RAS/RAF/MEK/ERK pathway, which is known to directly activate PI3K and cause cross-inhibition and cross-activation of PI3K pathways[70]. Other molecular targets, such as HER-2, VEGF and EGFR, have also been associated with PI3K pathways in cancers. The combination of PI3K pathway inhibitors with other targeted inhibitors has shown promising results in bypassing resistance mechanisms in many cancers, although their clinical effects are still contradictory. Our subgroup results showed that dual-targeted therapies that included a PI3K inhibitor showed inconsistent results in the PFS compared with single targeted reagents. The combination of VEGFR and mTORC1 targeted treatments showed no improvement in the PFS of patients, which may be due to the redundant angiogenic pathways or drug resistance, but novel PI3K inhibitors and mTORC2 inhibitors may help resolve this problem[71, 72]. These results may also be attributed to the failure to pre-select suitable patients by molecular analysis and to the unbearable toxic or side effects from dual-targeted therapies.

Our results showed no significant differences in OS between the experimental arm and the control arm. These results may be explained by the finding that most trials included in this analysis used PFS as the primary end point with a relatively short follow-up time, and many of them were phase II trials with a limited number of participants. Thus, the data were inadequate to detect differences in OS. Other factors, such as additional lines in the treatment arm and subsequent drug crossover, different combination therapies (chemotherapy or targeted therapy) with PI3K inhibitors, and the heterogeneity of cancer subtypes can all affect the results of OS. Therefore, the use of PFS instead of OS as an end point is adequate in PI3K pathway inhibitor trials.

We also analyzed the toxicity of PI3K pathway inhibitors compared with the controls. We found that toxicity is an important barrier to the use of these reagents in clinical settings. A meta-analysis conducted by Kenya on everolimus in hepatocellular carcinoma reported that everolimus significantly increased the incidence of liver injury (higher alanine aminotransferase), stomatitis, anaemia, hyperglycaemia and pneumonitis[73]. Hess compared two doses of temsirolimus (a mTOR inhibitor) with investigator choice in mantle cell lymphoma and found that the higher dose of temsirolimus significantly improved the PFS compared with the tumour response rate of the lower dose of temsirolimus but significantly increased the number of grade 3 and 4 adverse events[74]. After a rough review of the studies included in our analysis, it was found that the toxicity that induced trial discontinuation was obviously higher in the PI3K pathway inhibitors arms (16.7%) than in the control arms (9.8%). Serious toxicity may prevent PI3K pathway inhibitors from achieving their effective anti-tumour effects, which thus weakens their effects and limiting their broad use in clinical settings. Therefore, the circumvention of this problem is crucial for PI3K inhibitors.

Our meta-analysis has some limitations. Differences in the treatment line, the combination of chemotherapeutic regimens, dose and treatment circles among these trials were difficult to fully balance, although we performed some subgroup analyses. For some types of cancers, such as endometrial cancer, glioblastoma and melanoma, the power of the analysis of the effect of PI3K pathway inhibitors was insufficient because only one trial was available for each of these cancers. Lastly, although all studies included in this analysis were randomised controlled trials, most of them were phase II trials with a limited number of participants, and the assessment criteria and methods differed among trials, which are also limitations of our study.

## Conclusions

Our meta-analysis results suggest that the addition of PI3K pathway inhibitors to the therapy regimens for advanced solid tumours significantly improved the PFS, especially among patients with breast cancer and neuroendocrine tumours and those with PI3K mutations. However, this study was unable to observe improvements in the OS and ORR as a result of PI3K pathway inhibitors. Considering the side effects of PI3K pathway inhibitors when these drugs are used, the risk-benefit analysis must be carefully performed. In the future, more studies that are focused on selected types of cancers will be required to identify suitable patients who will benefit the most from therapies with PI3K pathway inhibitors.

## Supporting information

S1 Fig Forest plots of odds ratio (OR) for adversed event induced the study discontinue. Experimental arm included different kinds of PI3K/AKT/mTOR inhibitors. The random-effects model was used.
(TIF)

S1 Table PRISMA checklist. (DOC)

S2 Table Supplement information of the included studies. Abbreviation: NR: not reported; NA: not available; PFS: progression-free survival; OS: overall survival; IFN: interferon; ORR: objective response rate; TTP: time to progression; CBR: clinical benefit rate; DCR: Disease control rate; EGFR: epidermal growth factor receptor; VEGFR: vascular endothelial growth factor receptor; mTORC1: mammalian target of rapamycin complex 1. * Reported a different trial by the previous author in the same year. # Reported more than one comparation in a trial. Lowercase letter a, b,c means different trial arm in the same study.
(XLS)
