# Supplementary figures and images for: Efficacy of PI3K/AKT/mTOR pathway inhibitors for the treatment of advanced solid cancers: A literature-based meta-analysis of 46 randomised control trials
Source: PLoS One. 2018 Feb 6;13(2):e0192464. doi: 10.1371/journal.pone.0192464 (PMC5800666; doi:10.1371/journal.pone.0192464)

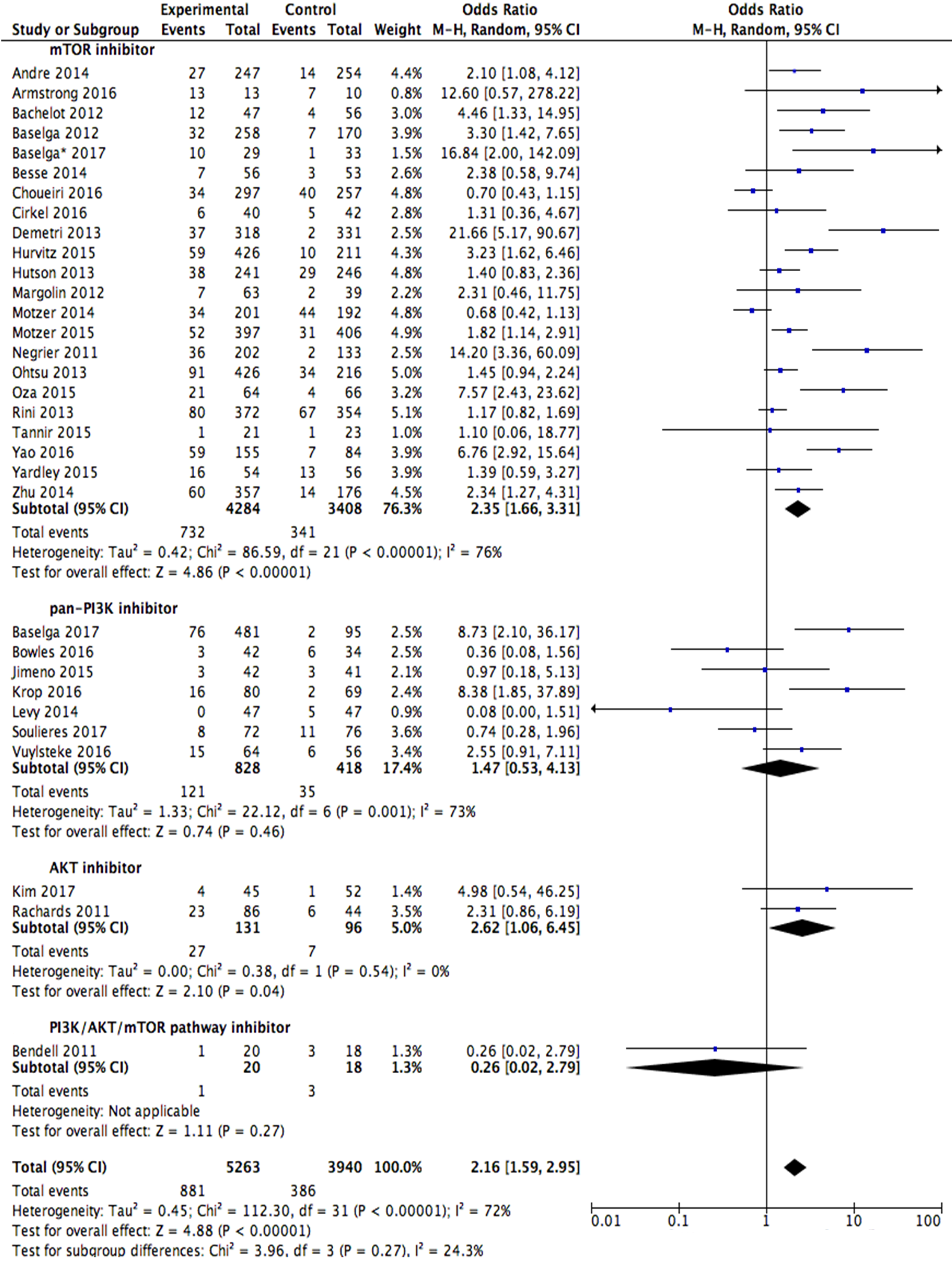

Supplement: S1 Fig — Experimental arm included different kinds of PI3K/AKT/mTOR inhibitors. The random-effects model was used. (TIF) [file pone.0192464.s001.tif]
